# sgnesR: An R package for simulating gene expression data from an underlying real gene network structure considering delay parameters

**DOI:** 10.1186/s12859-017-1731-8

**Published:** 2017-07-04

**Authors:** Shailesh Tripathi, Jason Lloyd-Price, Andre Ribeiro, Olli Yli-Harja, Matthias Dehmer, Frank Emmert-Streib

**Affiliations:** 1 Predictive Medicine and Data Analytics Lab, Department of Signal Processing, Tampere University of Technology, Tampere, Finland; 2 Department of Biostatistics, Harvard T.H. Chan School of Public Health, Harvard University, Boston, USA; 3 Laboratory of Biosystem Dynamics, Department of Signal Processing, Tampere University of Technology, Tampere, Finland; 4 Institute for Theoretical Informatics, Mathematics and Operations Research, Department of Computer Science, Universität der Bundeswehr München, Munich, Germany; 5 Institute of Biosciences and Medical Technology, Tampere, Finland; 6 Computational Systems Biology, Department of Signal Processing, Tampere University of Technology, Tampere, Finland

**Keywords:** Gene expression data, Gene network, Simulation

## Abstract

**Background:**

sgnesR (Stochastic Gene Network Expression Simulator in R) is an R package that provides an interface to simulate gene expression data from a given gene network using the stochastic simulation algorithm (SSA). The package allows various options for delay parameters and can easily included in reactions for promoter delay, RNA delay and Protein delay. A user can tune these parameters to model various types of reactions within a cell. As examples, we present two network models to generate expression profiles. We also demonstrated the inference of networks and the evaluation of association measure of edge and non-edge components from the generated expression profiles.

**Results:**

The purpose of sgnesR is to enable an easy to use and a quick implementation for generating realistic gene expression data from biologically relevant networks that can be user selected.

**Conclusions:**

sgnesR is freely available for academic use. The R package has been tested for R 3.2.0 under Linux, Windows and Mac OS X.

**Electronic supplementary material:**

The online version of this article (doi:10.1186/s12859-017-1731-8) contains supplementary material, which is available to authorized users.

## Background

Networks provide a statistical and mathematical framework for the general understanding of the complex functioning of biological systems because the causal relationship between different entities, such as proteins, genes or metabolites, defines how a cellular system functions collectively. This leads to an emergent behavior, e.g., with respect to phenotypic aspects of organisms [1–4]. Unfortunately, understanding of the system’s functioning of a cell is not an easy task and one reason for this is that the causal inference of gene network itself is a formidable problem [5, 6]. For this reason, we provide the R package sgnesR (Stochastic Gene Network Expression Simulator in R). Specifically, sgnesR can be used to generate biologically realistic gene expression data based on an underlying gene regulatory network that can be used to test network inference methods qualitatively. In this way an inferred network can be compared with the known *true* gene regulatory network, which is for most real biological systems unknown requiring the usage of approximations, e.g., by using transcriptional regulatory networks or protein interaction networks [7]. Overall, our package sgnesR enables the quantitative estimation of important statistical measures, e.g., the power, false discovery rate or AUROC values of such inferred networks. Furthermore, the resulting gene expression profiles can be itself of use for instance for comparison with real measurements of gene expression values for the identification of model parameters.

In general, the simulation of biologically realistic gene expression values is a challenging task because it requires the specification of transcription and translation mechanisms of biological cells, which are far from being understood in every detail. Specifically, there are two major components that need to be defined for the simulation of such a process. The first relates to the connection structure among the genes and the second to the parameter values of the modeling equations. The connection structure corresponds to the regulatory network which defines which genes control the expression of other genes. Our package sgnesR allows the usage of previously inferred biological networks or the usage of artificially simulated networks. For the identification of the parameters of the modeling equations of the transcription and translation processes values can be sampled from plausible distributional assumptions.

In the following, we discuss some existing methods that have been proposed and implemented for the simulation of gene expression data. An overview of these simulation methods for which software implementations are available is shown in Table 1. One of the most widely used methods is *syntren* [8]. Syntren uses an interaction kinetics model based on the equations of Michaelis-Menten and Hill kinetics. In contrast, *netsim* applies a fuzzy logic for the representation of interactions for a given topology of a gene regulatory network and differential equations to generate expression data [9]. Despite these differences, both simulation methods aim at emulating a biological model of transcription regulation and translation. A completely different approach is used by *GeneNet* [10]. This method samples network data from a Gaussian graphical model (GGM) for a given network structure. A similar approach is used in [11].

**Table 1:** A list of network sampling and simulation methods

Methods *⇓*∖ Features ⇒	Method-based on	Input	Output
sgnesR (SGN sim [13])	A set of biochemical reactions where transcription and translation of genes and proteins are modelled as multiple time delayed events and their activities are modelled by a stochastic simulation algorithm (SSA) [20]	S4 data object with a network of *igraph* class.	S4 data object which consists expression data matrix.
AGN [25]	Set of biochemical reactions in the form of a network, simulation of the kinetics of systems of biochemical reactions based on differential equations.	SMBL	Text file
GenGe [26]	Non linear differential equation system where degradation of biological molecules are modelled by a linear or Michalies-Menten kinetic and translation is described by a linear kinetic law by using several global and local perturbation parameters.	SMBL	Text file (numeric values).
GRENDEL [27]	A set of differential equation system uses hill kinetics based activation and repression functions for the transcription rate law.	SMBL	Text file (numeric values)
NetSim [9]	Differential equations are used to to model the dynamics of transcription and degradation along with the integration of fuzzy logic in order to define the complex regulatory mechanism	adjacency matrix with other parameters	list object in R
RENCO [28]	Uses pre defined network topology or generates topologies to model ordinary differential equations and use Copasi for simulating expression data.	Text file	Text file
SynTReN [8]	The interactions of a network uses non-linear functions based on Michaelis-Menten and hill enzyme kinetic equations to model gene regulation	Text file	Text file

Our R package *sgnesR* provides an easy-to-use interface to simulate gene expression data generated by the stochastic simulation algorithm (SSA) [12, 13]. That means a gene regulatory network is modeled whose activation patterns are defined by the transcription and translation which are modeled as multiple time delayed events. The delays itself can be drawn from a variety of distributions and the reaction rates can be determined via complex functions or from physical parameters. The original implementation of the ’Stochastic Gene Networks Simulator’ (*SGNSim*) algorithm [13] is available in C/C++. However, by providing the R interface *sgnesR*, it is possible to perform all relevant analysis steps, e.g., for testing network inference methods or for investigating pathway methods, within the R environment. This is not only convenient but leads to a natural integration of all parts making the overall analysis reproducible in the most straight forward way [14]. In addition, our package *sgnesR* allows selection capabilities for various biological and artificially simulated gene regulatory networks that can be used as realistic wiring diagrams for the interactions between genes.

The paper is organized as follows. In the next section we describe our gene expression simulator *sgnesR* in detail and present some working examples. These examples will demonstrate the capabilities of *sgnesR*. The paper finishes with a summary and conclusions.

## Implementation

**Fig. 1:**
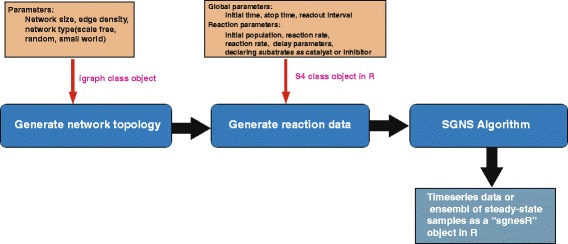
A flow chart of R implemented interface of Stochastic Gene Networks Simulator

In this section, we provide a description of the organizational structure corresponding to the workflow of the *sgnesR* package and its components. Schematically, the overview of the workflow is shown in Fig. 1. The first step consists in specifying the network topology. Here the user has two choices: A) use an external network or B) generate a simulated network. For B) we are using the *igraph* package in R. The *igraph* package provides a comprehensive set of functions that allows to generate or create several types of networks and compute several network related features; for the visualization of networks see [15]. A user can easily generate a network forming the connections for a set of reactions as the input of the SGNS algorithm [13]. Alternatively, a user can select biological networks as input as provided by public databases, e.g., [16, 17]. For convenience, we provide two biological networks in the sgnesR package. The first one is a transcription regulator network of E. coli [18] and the second a subnetwork of the human signaling network [19].

In addition to the specification of a graph topology, the assignment of initial populations of RNAs and proteins for each node and the activation or suppression indicator for each edge of the network are initialized in the first step of the *sgnesR* package. In the following, a brief description of the generation of the set of reactions from a network topology is provided.

Suppose, we have a network consisting four genes (nodes) A, B, C and D. Their interactions are described as follows:



In order to represent the following network topology as a set of chemical reactions we assume that each node is represented by a promoter, an RNA and a protein product. For example the node A is represented as ProA (promoter), RA (RNA) and PA (protein produce). In the following example below, A interacts with three nodes so A has three different promoter sites where the protein products of different genes (B, C and D) bind to activate or suppress the expression of A. The set of reactions are divided into three sections as follows:

1. Reactions for translation and degradation for each gene: In this step, three steps of reactions describe the translation of RNAs of each node into the protein products and the respective decay of each RNA and protein product. The example is shown below.

2. Binding-unbinding reactions: This set of reactions describe the binding of protein products of interacting genes to the promoter sites of interacted gene. In the given example, genes B and C activate and gene D suppress the expression of gene A so the protein products of B, C, and D interact with their respective promoter sites ProA.NoB, ProA.NoC and ProA.NoD in gene A and form intermediary products ProA.B, ProA.C and ProA.D. These intermediary products take part in the transcription process of the gene A. The gene D suppresses the expression of gene A, in this process an intermediary product of suppressor gene (ProA.D) is formed by Protein product of D (PD) by binding to the promoter site of the gene A (ProA.NoD). The intermediary product of suppressor gene D (ProA.D) does not allow to express gene A, therefore avoids the transcription process and releases after sometime. The example of binding and the unbinding of proteins to promoters sites is shown below.

3. Transcription reactions: This is a set of reactions of the transcription process of the gene to which all possible combinations of the intermediary products of the activators of the genes contributes to the expression of gene A. In this example, the two activators B and C can have three possible choices to contribute to the expression of A in which the intermediary product of only B, intermediary product of only C and intermediary products of both B and C contribute to the expression of the RNA of gene A. The example reaction is shown below:

These three sets of reactions along with other reaction parameters are passed to the SGNS algorithm to generate the expression profiles for the different genes. The additional reaction parameters needed are the initial population, reaction rates and delay parameters which are described in the following:

- Initial populations: The initial population of parameters assigns the initial values of promoters, RNAs and proteins for all the genes in the network.
- Reaction rates: The reaction rate parameter assigns values for *reaction-rate* to different reaction types for translation and degradation reactions as translation rate, RNA degradation rate and protein degradation rate. For binding and unbinding reactions it assigns binding and unbinding rates and for transcription rates it assigns transcription rate.
- Delay parameters: The delay parameter assigns a delay time for RNAs and proteins in translation and degradation reactions to be released at a certain time point. Also, the promoter delay is assigned to the products of transcriptions reactions to be released at a certain time point.

The sgnesR package provides two options to obtain the expression profiles of different genes as either time series data or steady-state values. The time series data is a set of expression values of different genes between the different time points of starting time and end time of reactions which are captured at fixed time intervals. The steady state values are final expression values of different genes at the end of the reaction. Furthermore the sgnesR packages allows to repeat the simulation of a input network *n* times and generates this way an ensemble of steady-state expression values of sample size *n*.

## Results and discussion

In this section, we present some working examples for the usage of our package *sgnesR*. These examples demonstrate some of the available features of its capabilities. The *sgnesR* package provides options to apply various parameters using base R functions and a variety of network topologies, based on several network features as parameters for generating simulated data. Further parameters are assigned to each reaction by defining two data objects of the “rsgns.param” and “rsgns.data” class. These are defined as follows.

- “rsgns.param”: This class defines the initial parameters which include “start time”, “stop time” and “read-out interval” for time series data.
- “rsgns.data”: The class defines a data object for the input which includes the network topology and other parameters such as the initial populations of RNA and protein molecules of each node/gene, rate constants, delay parameters and initial population parameters of different molecules.
- “rsgns.waitlist”: This class defines the molecules placed in a waiting list and to be released a specific number of molecules at a particular time during the reaction. This class includes “nodes”, “time”, “mol” and “type” for time series data.

### R functions for generating data from a given network

- *getreactions* : This function generates an object of class “rsgns.reactions” which contains a set of reactions, their initial values and the wait-list of reactions. This object can be supplied to the SGNS API for generating gene expression data. The “rsgns.reactions” object is a list containing six components which are “population”, “activation”, “binding_unbinding”, “trans_degradation” and “waitlist”. Each component of the list is a matrix object and user can modify those reaction parameters depending on the requirements before passing it to “rsgns.rn” function as an input.
- *rsgns.rn*: This function is an interface to the SGNS API for simulating timeseries data. A user can either provide a “rsgns.reactions” class object directly to the function or the “rsgns.data” class object to receive the output. There are further options available to tune the reaction parameters. The function itself returns a “sgnesR” class object which contains the generated expression data, the input network and the reaction kinetics information.
- *plot.sgnesR*: This function provides different options to visualize the expression profiles. The function has two major options available. The first one is to visualize the expression values in terms of RNA numbers at different time points and the second option is to visualize the distribution of RNA numbers for different nodes/genes at different time points or the sample-distribution of an ensemble of steady state values.

### Generating time series data from a scale-free network

**Fig. 2:**
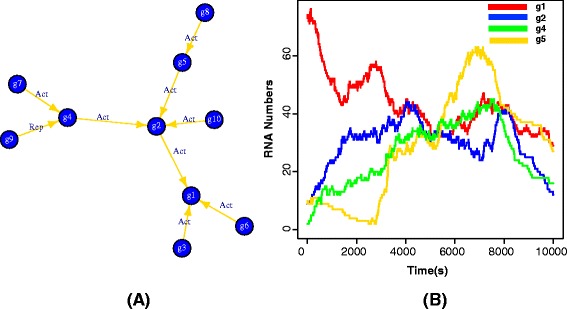
A plot of sample network and the expression values at different time points of different nodes from the simulation. **a** The input network **b** Expression values of genes which show incoming edges



The first example we demonstrate how to use sgnesR package to generate time series data from a scale-free network. The code for this is presented in Example 1. For reasons of simplicity, in this example we do not consider delay parameters for the translation and transcription processes (see Example 2 for an extension). The visualization of the network and the generated expression values are shown in Fig. 2.

### Generating time series data from a scale-free network with delay parameters

In Example 2 we provide a working example to generate time series data from a scale-free network with delay parameters. That means we are assigning delay parameters for the translation reactions of the RNA delay and the protein delay and in transcription reactions for a promoter delay. The user can assign delay parameters chosen from a Gaussian distribution with different mean values and variance. Further choices are delay functions such as a gamma distribution or an exponential function for the delays. However, for simulating real biological gene expression data it is preferable to use the “gamma” function to assign delays [20].



### Generating steady-state samples of expression values from an Erdos-Renyi network

**Fig. 3:**
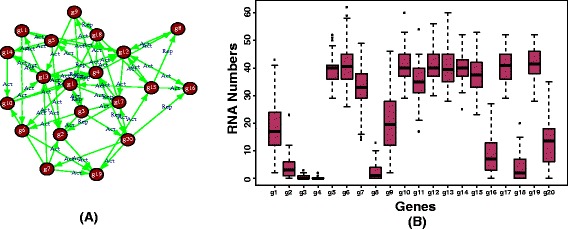
A plot of input network and the the distribution of expression values of different samples from the simulation. **a** The input network **b** Distribution of expression values of genes for different samples



Here ’steady-state samples’ means ’asymptotic samples’ in the sense that we run our simulations until the expression values of the genes reach constant values where a further continuation of the simulations lead to no further changes of expression values of the molecules. Example 3 provides a working example to demonstrate the usage of our package. The visualization of the results of the network and the distribution of the ensemble of generated expression profiles is shown in Fig. 3. We want to remark that the ’sample’ option for the function ’rsgns.rn’ means that the simulations are repeated n times, as defined by the value of ’sample=n’, by using the same initial values of all parameters. In case the user wants to use different initial values, then ’sample=1’ needs to be used and an explicit loop over ’rsgns.rn’ needs to be carried out.

### Generating time series data from a known set of equations

In this example we demonstrate how to use sgnesR package to generate time series data from a user defined set of reactions. The code for this is presented in Example 4. This example is based on the toggle switch reactions without cooperative binding. The purpose of this example is to simulate a set of reactions when we know the information of promoter regions along with RNA and protein binding information. Suppose the equations are described as follows:

1. ProA + *Ind –[0.002]– > A + ProA
2. ProB + *Ind –[0.002]– > B + ProB
3. A –[0.005]– >
4. B –[0.005]– >
5. A + ProB + *ProA –[0.2]– > ProB.A
6. B + ProA + *ProB –[0.2]– > ProA.B
7. ProB.A –[0.01]– > ProB + A
8. ProA.B –[0.01]– > ProA + B
9. ProB.A –[0.005]– > ProB
10. ProA.B –[0.005]– > ProA

### Application in network inference

**Fig. 4:**
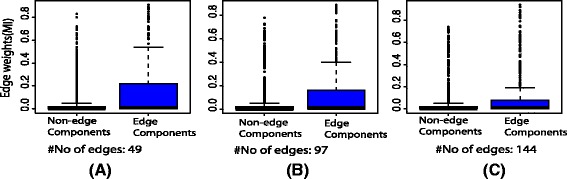
The distribution of edge-weights of gene-pairs of non-edge components and edge components of inferred networks using *BC3NET* from the simulated expression profiles of artificial networks generated by *sgnesR*. In (**a**), (**b**) and (**c**) example networks are shown that have a different number of edges

**Fig. 5:**
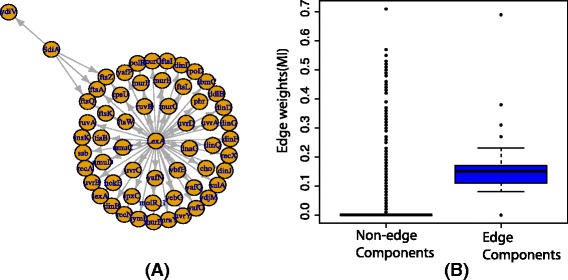
**a** A subnetwork of transcription regulatory network of *ecoli* used to simulate expression profiles using *sgnesR*. **b** The distribution of edge-weights of gene-pairs of non-edge components and edge components of inferred network using *BC3NET* from the expression profiles of *ecoli* subnetwork generated by *sgnesR*

In this section, we present two examples to generate expression profiles and the inference of networks from the expression profiles using BC3NET [22]. BC3NET is a network inference method based on the ensemble of inferred networks by assigning an edge for a gene-pair if at least one of these two genes show maximal mutual information with respect to all other genes [23]. For simulation, we chose two types of networks the first one are the scale-free artificial networks with 50 nodes and edges of different edge densities. The second network is a subnetwork of *ecoli* transcription regulatory network [24] which contains 59 nodes and 60 edges. The subnetwork is shown in Fig. 5(a). The generated expression profiles of *ecoli* transcription subnetwork are based on hypothetical promoter regions where an RNA molecule of a gene binds to a hypothetical promoter region of another gene if there is an edge exist between them. The other parameters of the reactions are hypothetical assumptions for the reactions. The details of these parameters and generation of expression profiles are provided in the *supplementary R* file (ecolisim_script.R). In the first step, we generate expression profiles of artificial networks and *ecoli* subnetwork using *sgnesR*, in the second step we used expression profile for inferring networks using *BC3NET*. For all three types of artificial networks, we repeat simulation 20 times. For each simulation step, the mutual information is calculated between all pairs of nodes using *BC3NET* which assigns weights to all pair of nodes. In this simulation, we highlight the distribution of weights of gene-pairs which are connected by edges and gene-pairs which are not connected with each other (non-edge). The results are shown in Fig. 4. Similarly, we generate expression profiles using the *ecoli* network and inferred the network using *BC3NET*. The distribution of weights of gene-pairs which are connected by edges and gene-pairs which are not connected with each other (non-edge) are shown in Fig. 5(b). In these examples, we clearly see that the *BC3NET* assigns higher weights by computing mutual information of expression profiles to the pairs of nodes for edge components compare to the non-edge components of simulated networks and *ecoli* subnetwork. Similarly, the other measures can be used to evaluate the performance of different network inference methods.

### Computational complexity

Overall, the computational complexity of the algorithm depends on the edge density of the used network and specifically on the in-degree of each node. However, for networks with up to ∼1000 genes the package generates rapid results. A practical overview of the run time of our sgnesR package is shown in Table 2. The average run time is shown in seconds for different network sizes. We repeated the analysis 10 times for each network size shown in the table.

**Table 2:** Estimated time by *sgnesR*, in seconds for different type of networks

Network size	Average edge size	Maximum degree (Average)	Average run time (seconds)
20	21.9	6.4	0.25
50	55.4	9.0	0.42
100	114.0	10.7	1.92
150	165.2	12.4	7.77
200	227.1	12.5	14.10
500	560.9	15.4	116.31
1000	1110.8	17.8	391.04

We would like to remark that the theoretical computational complexity of the implementation of the SGNS algorithm has a formal time complexity of *O*(*T* *R*∗(*D* log*R*+ log*W*)). Where T = simulation time, R = number of reactions, D = max degree in propensity update dependency graph between reactions, W = max wait list size. However, our sgnesR package contains an additional layer of complexity consisting of the automatic generation of all reaction equations for a given network topology.

## Conclusions

In this paper, we described the R implementation of the *sgnesR* (Stochastic Gene Network Expression Simulator) package. The main objective of the sgnesR package is to utilize the applicability of gene expression simulations, e.g., for validating the performance of network inference methods [5, 6]. The *sgnesR* package allows an easy-to-use interface for the simulation of gene expression profiles from a given network structure. A user can easily either utilize a given biological network or generate a topological structure of different network types for which reaction parameters are specified in correspondence to given constraints. In our package the reaction parameters can be modeled and used in a very flexible manner, e.g., with respect to the underlying parameter distributions. The resulting gene expression data can be either obtained as time series data for user defined sampling time steps or as steady-steady data.

## Availability and requirements

**Project name:** *sgnesR*

**Project home page:** “Package is currently available on: https://github.com/shaileshtripathi/sgnesR”

**Operating system(s):** Windows, Linux, OS X

**Programming language:** R, C

**License:** Free

## Additional file

Additional file 1
*ecolisim_script.R*, example R script to simulate expression profiles and the inference of network from the simulated profiles using *BC3NET*. (R 2 kb)

## Abbreviations

AUROC: Area under receiver operator characteristics (ROC) curve
sgnesR: Stochastic gene network expression simulator in R
SSA: Stochastic simulation algorithm
